# “Medicine food homology” plants promote periodontal health: antimicrobial, anti-inflammatory, and inhibition of bone resorption

**DOI:** 10.3389/fnut.2023.1193289

**Published:** 2023-06-15

**Authors:** Shanlin Qu, Shuo Yu, Xiaolin Ma, Rui Wang

**Affiliations:** ^1^Hospital of Stomatology, Jilin University, Changchun, China; ^2^Jilin Provincial Key Laboratory of Tooth Development and Bone Remodeling, Changchun, China

**Keywords:** medicine food homology plants, periodontitis, antibiosis, virulence factor, anti-inflammatory, bone resorption

## Abstract

“Medicine food homology” (MFH) is a term with a lengthy history. It refers to the fact that a lot of traditional natural products have both culinary and therapeutic benefits. The antibacterial, anti-inflammatory and anticancer effects of MFH plants and their secondary metabolites have been confirmed by numerous research. A bacterially generated inflammatory illness with a complicated pathophysiology, periodontitis causes the loss of the teeth’s supporting tissues. Several MFH plants have recently been shown to have the ability to prevent and treat periodontitis, which is exhibited by blocking the disease’s pathogens and the virulence factors that go along with them, lowering the host’s inflammatory reactions and halting the loss of alveolar bone. To give a theoretical foundation for the creation of functional foods, oral care products and adjuvant therapies, this review has especially explored the potential medicinal benefit of MFH plants and their secondary metabolites in the prevention and treatment of periodontitis.

## 1. Introduction

A multifactorial microbial infectious disease known as periodontitis is characterized by gingival bleeding, swelling, attachment loss and bone absorption, as well as other destructive changes in the periodontium that finally lead to tooth loss, decreased chewing function, changes in food intake or eating habits and a reduction in the quality of the patient’s life (1). Simply put, the intricate interaction between subgingival microorganisms and the host immune system leads to periodontitis. The incidence of periodontitis has increased by 83.4% in the last 10 years, and population growth and aging are raising the disease’s global burden (2). In addition, studies have revealed that periodontitis is linked to several different health conditions throughout the body, including atherosclerosis (3), diabetes (4), cancer (5) and respiratory problems (6).

Traditional Chinese medicine has used the term “MFH” since the beginning of time. Eating when one is hungry is treated as taking medicine and given to the patient as medicine, which is a statement that reflects the concept of “MFH” made in “*Huang Di Nei Jing*” (7). The term “MFH plants” alludes to the fact that many Chinese medicines double as food and medication. In addition, many traditional Chinese medicines can also be used in functional foods that are suitable for specific groups. Although functional food cannot replace medicine, it can regulate human bodily functions and help maintain and promote human health (8). Furthermore, many secondary metabolites found in MFH plants have been demonstrated to have anti-inflammatory (9), antibacterial (10) and anticancer (11)effects, indicating the potential use of MFH plants in disease prevention and medical care.

**Figure 1:**
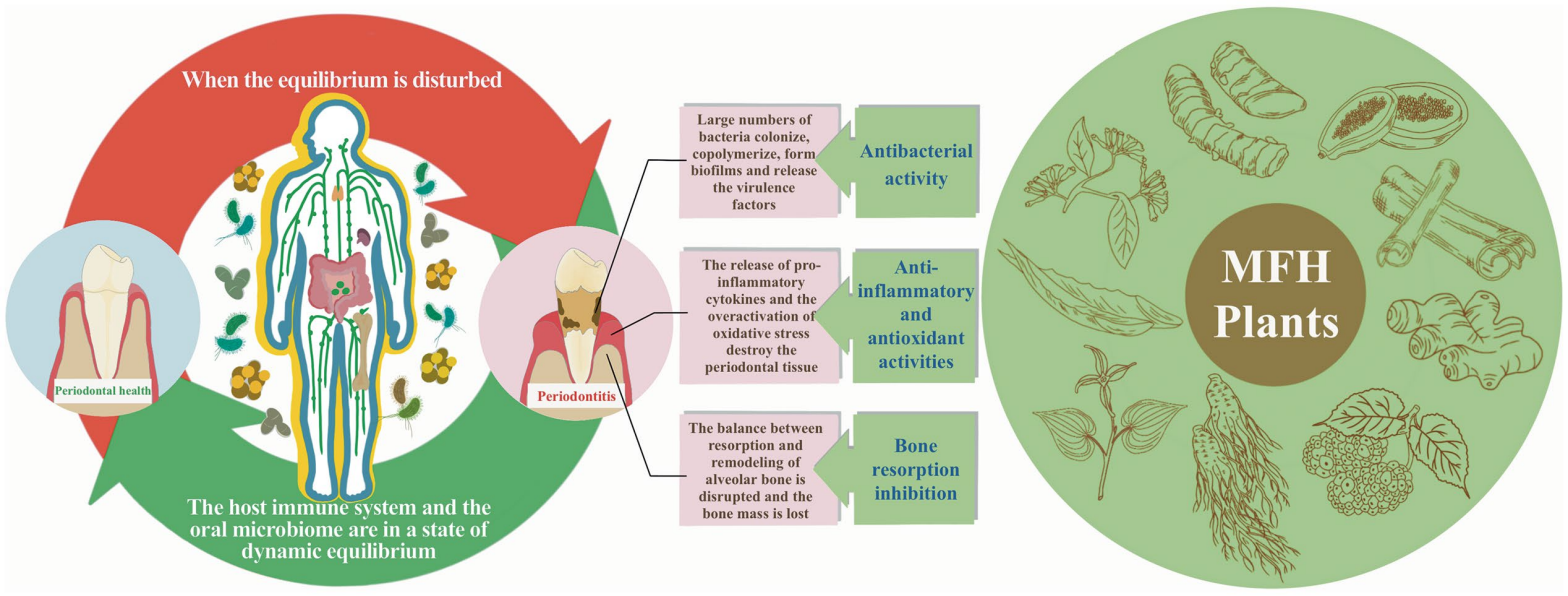
The etiology and pathogenic process of periodontitis as well as MFH plants’ anti-periodontitis effects.

The idea of “food therapy” has recently been used in the preservation of periodontal health. Several studies have noted that the body can get anti-inflammatory and antioxidant substances from the diet, like polyphenols and flavonoids, that the body cannot generate on its own (12). Several MFH plants are now useful against periodontitis in a variety of ways, including antibacterial action against periodontal pathogens, decreasing host inflammatory response and improvement of alveolar bone loss (Figure 1). In order to offer a theoretical framework for the use of pharmacological and food homology in periodontitis, the mechanism and research progress of MFH plants and their secondary metabolites against periodontitis were categorized and explained in this paper.

## 2. Pathogenic mechanism of periodontitis

The most prevalent form of bacteria in the oral microenvironment is plaque biofilm. It is primarily present on the outside of oral soft and hard tissues or prostheses and is made up of a very complex extracellular polymeric matrix community. Subgingival plaque is a crucial contributor to the etiology of periodontitis and is distinguished from supragingival plaque by the gingival margin. The rich substances in the gingival fluid serve as the essential nutrient supply for bacteria, and the gingival sulcus offers a rather stable dwelling environment for bacteria. The amount and makeup of the bacterial population in plaque vary dynamically depending on the host’s state of health or sickness so that the environment in plaque is not constant (13). In contrast to the healthy condition, the subgingival bacterial community will be much more diverse and abundant in diseased periodontal tissue. The proportion of some populations, such as those pathogens linked to periodontitis, will also be relatively higher (14). Studies have shown that *Porphyromonas gingivalis* (*P. gingivalis*), *Tannerella forsythia*, *Treponema*, *Fusobacterium nucleatum* (*F. nucleatum*), *Prevotella intermedia* (*P. intermedia*), *Actinobacteria*, *Pseudomonas aeruginosa* (*P. aeruginosa*) and so on (15–17), has been confirmed to have a strong correlation with the onset of periodontitis. *P. gingivalis*, *Tannerella forsythia*, and *Treponema*, also known as the red complex, have the strongest correlation with periodontitis (18). Even at low concentrations, these bacteria can cause inflammatory reactions in the periodontal tissues, yet these reactions are consistent with the tissues’ defensive capabilities, allowing the periodontal tissues to continue to function normally (19). However, when this balance is upset by some factors, such as poor host oral hygiene, bad habits like smoking, or systemic diseases, the host periodontal tissue’s defensive capability is significantly diminished, which promotes the growth of some periodontal pathogens and makes them absolutely dominant in quantity and abundance, as well as has an impact on the host-microbial crosstalk (20). The microbiota is encouraged to change into a more pathogenic state when the host’s periodontal tissues experience an inflammatory reaction (21). Additionally, some bacterial structural elements, biomacromolecules and byproducts are cytotoxic and aid in bacterial colonization, copolymerization, biofilm formation, nutrient uptake, host cell invasion and escape ability, among other processes that are thought to participate in the inflammatory response of periodontitis (22–24). According to certain studies, a healthy, stable biological community is less likely to cause an inflammatory response in periodontal tissue than an ecologically unbalanced population rich in virulence factors (25). Moreover, the etiology of systemic disorders may be influenced by the ectopic colonization or circulation of periodontal bacteria, virulence factors, or specific mediators (26, 27).

Although bacteria are the initial causes of periodontal tissue inflammation and injury, they are not the primary component driving the development of the disease (28). When some cells and receptors in the host periodontal tissue recognize and kill invading pathogens, they also cause damage to the periodontal tissue. The equilibrium between tissue breakdown and regeneration is upset when the immune response is overactive and activates to levels above what is considered normal. This intensifies organizational destruction. Host immunity consists mainly of congenital and acquired immunity (29). Bacterial metabolites stimulate epithelial cells to produce pro-inflammatory cytokines and neurons release neuropeptides to induce vasodilation and promote neutrophil migration to the inflammatory site of the periodontal tissue to resist pathogen invasion. If the infection is not eliminated, it can lead to early lesions, mainly characterized by gingival hyperplasia, bleeding and increased crevicular fluid flow. Innate immunity progressively changes into acquired immunity as the severity of gingivitis rises, and this process is mediated by macrophages, plasma cells and T and B lymphocytes. Blood circulation is hampered and collagen fibers keep dissolving. Mild gingivitis now advances to moderate to severe gingivitis, and it also appears to change color and shape at this stage. Gingivitis develops into periodontitis as the inflammation progresses deeper into the deep tissues, leading to loss of attachment and dental bone resorption (30). In the aforementioned process, cytokine release has the potential to directly destroy periodontal tissues in an irreversible manner as well as indirectly destroy them by altering intracellular signaling and gene expression (31). Aside from killing harmful bacteria, the respiratory burst of neutrophils can result in excessive production of reactive oxygen species (ROS) and matrix metalloproteinases, which can cause an oxidative imbalance in periodontal tissues and permanent tissue deterioration (32). The many immune response mechanisms used by the various cell types in the periodontitis immune response intersect and overlap, eventually generating the immunity/inflammation cascade reaction process, which leads to the complexity and multi-level pathogenesis of periodontitis.

## 3. Dietary intervention in periodontitis

Many researchers have supported the fact that diet quality can either promote or inhibit periodontal health. A healthy plant-based diet decreased the incidence of periodontitis and was favorably correlated with antibody levels, according to a cross-sectional study (33). Anti-inflammatory diets may help people with periodontitis improve their periodontal condition and reduce tooth loss, according to some research (34, 35). Cross-sectional research of Hispanic or Latino populations between the ages of 18 and 74 years revealed that those who consumed more whole grains, fruits, and vegetables and less red or processed meat had lower incidences of severe periodontitis (36). Sugar, snacks, and fast food, especially those high in processed carbohydrates, may promote the development of periodontitis by affecting the biofilm formation in the mouth (37). The buildup of oral biofilms is not decreased by a healthy diet, which includes fruits, dietary fiber, vegetables, and dairy products, but a good diet can encourage the growth of non-pathogenic microbial communities by controlling oral microbial populations. For instance, in saliva samples from obese people, the Mediterranean diet decreased the relative abundance of *P. gingivalis*, *P. intermedia*, and *Treponema* but did not affect the makeup of the overall salivary microbial community (38). However, there are difficulties in determining a causal link between dietary variables and periodontitis from the above-mentioned cross-sectional investigations. Several theories on the connection between diet and periodontitis have also been advanced by researchers. The Western diet, which predominantly includes processed meat, butter, high-fat dairy products and so on, was only linked to an increased risk of periodontitis among obese individuals, according to a prospective study of 34,940 men (39). Systemic inflammation, insulin resistance, and metabolic disorders associated with obesity may be key factors in the increased risk of periodontitis (40). In addition, obesity is significantly associated with diabetes, and numerous studies have shown that diabetes has a significant bidirectional promoting relationship with periodontitis (41). As everyone knows, dietary intervention is one of the most fundamental forms of treatment for diabetes and obesity. Therefore, we hypothesize that dietary intervention influences periodontal health through many mechanisms and has the ability to prevent periodontal damage brought on by diabetes and obesity. In recent years, significant advancements have been made by MFH plants in the treatment and management of diabetes and its complicating disease (8). MFH plants also have great potential in the prevention and treatment of periodontitis which is one of the complications of diabetes. Its dual use as medicine and food makes it safe, widely available, low cost and remarkable effect, which gives a fresh concept and approach to periodontitis dietaryintervention.

## 4. Antibacterial effects and the inhibition of bacteria-related virulence factors of MFH plants and their secondary metabolites

Many secondary MFH plant metabolites or extracts have demonstrated direct antibacterial action against periodontal infections. The most popular evaluation index is minimum inhibitory concentration (MIC). The MIC is the minimum concentration of an antibacterial agent required to totally prevent test strains of organisms from growing visibly under tightly controlled *in vitro* circumstances. It serves as an indicator of how sensitive bacteria are to antibiotics (42). Table 1 displays the findings of the MIC analysis for the MFH plant extract, secondary metabolites and associated products. The main mechanisms by which MFH plants exert their antibacterial effects on bacteria include the breakdown of bacterial cell walls or membranes as well as interference with the synthesis of DNA and other vital biological macromolecules. The key bioactive components of MFH plants’ basic chemical structure are directly related to this inhibitory function.

**Table 1:** Antibacterial effects of MFH plants, secondary metabolites and related products.

MFH plants	Extracts, essential oils or secondary metabolites and derived materials	Bacteria	MIC	Ref.
Clove	Clove essential oil	*P. gingivalis*	6.25 μg/mL	(43)
	Eugenol	*P. gingivalis*	31.25 μM	
Ginseng	Ginsenoside Rh4	*P. gingivalis*	31.3 μg/mL	(44)
		*F. nucleatum*	16 μg/mL	
		*P. aeruginosa*	125 μg/mL	
	Ginsenoside Rk3	*P. gingivalis*	62.5 μg/mL	
		*F. nucleatum*	16 μg/mL	
		*P. aeruginosa*	125 μg/mL	
	Ginsenoside Rg5	*P. gingivalis*	16 μg/mL	
		*F. nucleatum*	16 μg/mL	
		*P. aeruginosa*	62.5 μg/mL	
	Ginsenoside Rh2	*P. gingivalis*	16 μg/mL	
		*F. nucleatum*	16 μg/mL	
		*P. aeruginosa*	62.5 μg/mL	
	Ginsenoside Rd	*P. gingivalis*	400 μM	(45)
Cinnamon	CBEO	*P. gingivalis*	6.25 μg/mL	(46)
	Cinnamaldehyde	*P. gingivalis*	2.5 μM	
Licorice	Glabridin	*P. gingivalis*	1.562 μg/mL	(47)
	Licochalcone A	*P. gingivalis*	1.562 μg/mL	
	Isoliquiritigenin	*P. gingivalis*	12.5 μg/mL	
Roselle calyx	Extract	*F. nucleatum*	7.2 mg/mL	(48)
		*A. naeslundii*	14.4 mg/mL	
		*A. actinomycetemcomitans*	28.8 mg/mL	
		*P. gingivalis*	7.2 mg/mL	
		*P. intermedia*	14.4 mg/mL	
Aloe	Fresh aloe gel	*A. actinomycetemcomitans*	50 μg/mL	(49)
		*P.gingivalis*	50 μg/mL	
		*S.mutans*	25 μg/mL	
	Extracts of *Rheum palmatum* root and *Aloe vera*	*P. gingivalis*	2 mg/mL	(50)
Mulberry	Resveratrol	*P. gingivalis*	78.12–156.25 μg/mL	(51)
Turmeric	Curcumin	*P. gingivalis*	62.5-125 μg/mL	(52)
Psoraleae	Psoralen	*P. gingivalis*	6.25 μg/mL	(53)
	Angelicin	*P. gingivalis*	3.125 μg/mL

Additionally, MFH plants and their secondary metabolites can reduce bacterial viability. Instead of immediately killing and suppressing bacteria under the sub-MIC concentration, MFH plants are more likely to influence bacterial copolymerization, adhesion, biofilm formation, signal transmission, and nutrition intake by decreasing virulence factors associated with their survival and invasion. A specific component or metabolite of the bacteria may be the source of the virulence factor they create. It can encourage the inflammatory response of periodontal tissue and ultimately result in tissue loss in addition to aiding bacterial survival (23). Table 2 displays the correlational researches of the MFH plant extracts, secondary metabolites and associated products inhibiting the virulence factors of *P. gingivalis*. *P. gingivalis*, a kind of gram-negative anaerobic bacteria, has been linked to the etiology of periodontitis, which is the primary subject of investigations on the virulence factors connected to periodontal infections at the moment. Animal models exposed to *P. gingivalis* develop inflammatory reactions and lose alveolar bone. The virulence factors present in *P. gingivalis* include pili, gingipains, outer membrane vesicles (OMVs) and hemagglutinin (56, 57). The *fimA* gene encodes the protein FimA, which can be polymerized to produce pili and is involved in bacterial invasion of host cells, copolymerization, colonization and biofilm formation (58). By destroying periodontal tissue’s constituents, gingipains, such as the two kinds of arginine-specific gingipains (RgpA, RgpB) and the lysine-specific gingipains (Kgp) encoded by *rgpA*, *rgpB* and *kgp*, can hasten the start and progression of periodontitis (59). The *P. gingivalis* hemagglutinin may bind heme and hemoglobin, which is helpful for bacterial survival and the spread of infection. The hemagglutinin-encoding genes, *hagA* and *hagB*, are also strongly linked to the development of biofilms (60). Furthermore, A variety of virulence factors are enriched in OMVs, which include outer membrane proteins, lipopolysaccharide (LPS), phospholipids, DNA and cytoplasm (61).

**Table 2:** The ingredients from MFH plants and their inhibitory mechanism against virulence factors of *P.gingivalis*.

Bioactive ingredient	Type of compounds	Source	Related mechanism	Ref.
Eugenol	Phenolic compounds	Cloves, Cinnamon	Inhibition of the expression of pili-related genes *fimA*, *hagA*, *hagB* and gingipains-related genes *rgpA*, *rgpB* and *kgp*.	(43)
Resveratrol	Phenolic compounds	Mulberry	Inhibition of bacterial degradation and adhesion of typeIcollagen; down-regulation of the expressions of *fimA II* and *IV* and gingipains-related genes *rgpA* and *kgp*.	(51, 54)
Curcumin	Phenolic compounds	Turmeric	Inhibition of gene expression of *fimA*, *hagA* and *hagB* and gingipains-related genes *rgpA*, *rgpB* and *kgp*, thereby inhibiting biofilm formation and reducing bacterial adhesion.	(52)
Petroselinic acid	Fatty acid	Fennel	Triggers the formation of OMVs-like particles that enrich RagA and RagB on the surface of bacteria, which can trigger explosive bacterial degradation and spillage of cell contents and DNA.	(55)
Ginsenoside Rd	Saponin	Ginseng	Reducing bacterial surface hydrophobicity and blocking the expression of virulence genes (*fimA* and *kgp*), which has an impact on bacterial adhesion, copolymerization, and biofilm formation.	(45)

### 4.1. Phenolic compound

**Figure 2:**
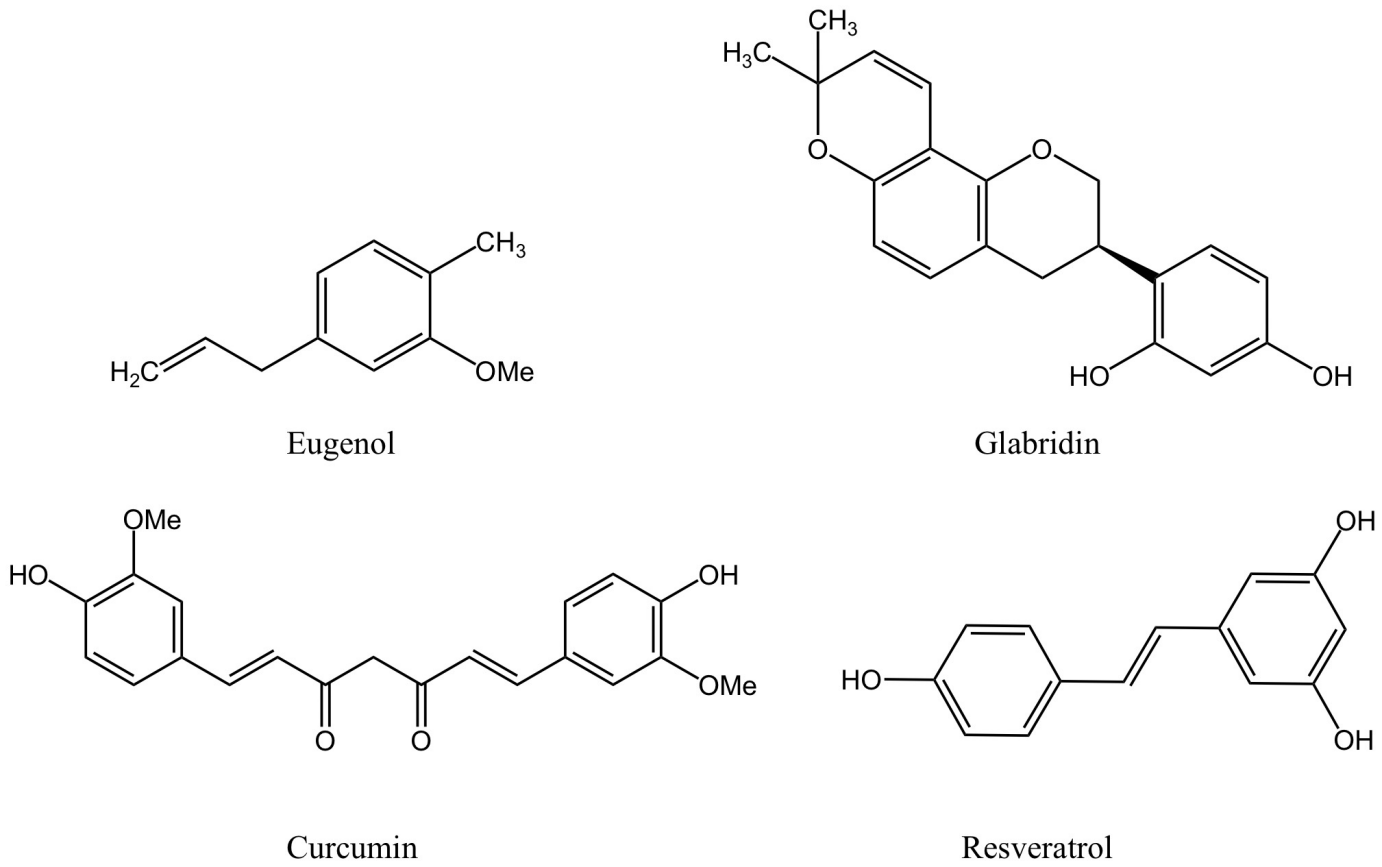
Phenolic compounds derived from MFH plants with antibacterial properties (created with KingDraw chemical structure editor).

Phenolic compounds are a typical class of active ingredient found in a variety of natural plants. They have at least one aromatic ring structure linking one hydroxyl group. Three categories are frequently used to categorize phenolic compounds: lignans and tannins, which are polyphenols; low phenolic oligophenols including flavonoids, stilbenes, and coumarins; and monophenols (62, 63). Numerous studies have established that phenolic compounds have an antibacterial impact, and some researchers have outlined the antibacterial mechanisms in detail. The current generally held beliefs can be summed up as follows: 1. The phenolic compounds alter cell permeability and causes leakage of cellular contents by interacting with bacterial cell walls or membranes, as well as surface proteins; 2. Reactive oxygen species (ROS) are produced by phenolic chemicals, which enhances oxidative stress in bacterial cells; 3. Phenolic compounds prevent the synthesis of bacterial-related biological macromolecules, which inhibits the bacteria’s development and metabolism; 4. Phenolic substances prevent DNA and ATP from being synthesized; 5. The production of proteins or virulence factors necessary for bacterial survival and biofilm formation are weakened by phenolic chemicals (64). The positions and amounts of the hydroxyl and methoxy groups (65–67), as well as the amounts and locations of hydrophobic substituents such as isopentenyl, alkylamino, and alkyl chains (68, 69), are all intimately related to the aforementioned activities. Figure 2 depicts the chemical composition of phenolic compounds found in MFH plants that have antibacterial properties.

The primary active component of the essential oils of several MFH plants, including *Syringa aromaticum* (cloves) and *Cinnamomum cassia* (cinnamon), is eugenol, a widely available monophenol molecule (70, 71). At MIC concentrations, eugenol can reduce the planktonic activity of *P. gingivalis* in a time-dependent manner. After being exposed to eugenol, *P.gingivalis* cells exhibited atrophic changes and severe cell membrane destruction, as revealed by a scanning electron microscope(SEM). In addition, leakage of nucleic acids and proteins within cells can be observed, indicating that eugenol interferes with cell membrane permeability and integrity. The hydrophobicity of eugenol and the impact of hydroxyl groups are closely related to the aforementioned actions (43). The extremely reactive hydroxyl groups in eugenol can create hydrogen bonds with a specific target enzyme of bacteria, which can disrupt the activity of the enzyme and result in a malfunction of the cell membrane system (72). Besides that, eugenol can indirectly inhibit the production of bacterial virulence factors by *P. gingivalis* by down-regulating the expression of the *fimA* genes, the hemagglutinin encoding genes *hagA* and *hagB* and the gingipain genes *rgpA*, *rgpB* and *kgp*, thereby affecting bacterial colonization, copolymerization, biofilm formation and nutrient uptake (43).

As a traditional Chinese medicine, *Glycyrrhiza glabra* (licorice) has high medicinal value in its dry roots and rhizomes and can also be used in the daily diet (73). The research demonstrated that the licorice extract’s flavonoid known as glabridin had a potent antibacterial effect against *P. gingivalis*. Furthermore, glabridin and the antibacterial peptide -poly L-lysine work together to kill bacteria (47). Glabridin has a high affinity for DNA gyrase and dihydrofolate reductase (DHFR) and can be utilized as inhibitors of two enzymes, even though the antibacterial mechanism is still unknown. Glabridin’s phenolic hydroxyl generates two hydrogen bonds with certain amino acids at DNA gyrase’s ATP binding site, preventing the synthesis of nucleic acids, and the affinity is higher than ciprofloxacin. Glabridin’s affinity for DHFR is primarily mediated *via* a hydrogen bond and other factors and is slightly weaker than trimethoprim’s (74).

*Fructus mori* (mulberry), the fruit clusters of the mulberry tree, are juicy and delicious fruits rich in the polyphenolic compound resveratrol (75). Resveratrol is a common stilbene compound, which can kill *P.gingivalis* and can be found that the size and shape of bacteria are damaged to varying degrees through SEM (51). Despite the fact that there is currently no agreement on resveratrol’s antibacterial capabilities (76), the double bond between the two benzene rings and the location and number of hydroxyl groups in the resveratrol structure, according to some researchers, may be important components in resveratrol’s ability to inhibit bacteria (77, 78). Other studies have demonstrated that following resveratrol therapy, the expression of the *P. gingivalis* pili-related genes *fimA II* and *IV* decreased by more than 50%, and the expression of the gingipains-related genes *rgpA* and *kgp* also decreased (51). Resveratrol can impair *P.gingivalis*’ adhesion to the basement membrane model and prevent *P.gingivalis* from degrading type I collagen in a dose/time-dependent manner (54).

*Curcuma longa* (turmeric) is an ancient Chinese medicinal plant of the ginger family, whose dried roots can be used in everyday cooking as well as for medicinal purposes. Curcumin is a phenolic compound extracted from turmeric (79). At concentrations below the MIC, curcumin can considerably limit the activity of six different strains of *P. gingivalis* and reduce its adhesion ability (52). Similarly, the researchers stressed the significance of aromatic hydroxyl groups in curcumin’s antibacterial effect (80, 81). Studies have shown that curcumin can downregulate the gene expressions of *fimA*, *hagA*, *hagB*, *rgpA*, *rgpB*, and *kgp*, inhibit the formation of *P. gingivalis* biofilm and reduce bacterial adherence (52).

### 4.2. Saponin

**Figure 3:**
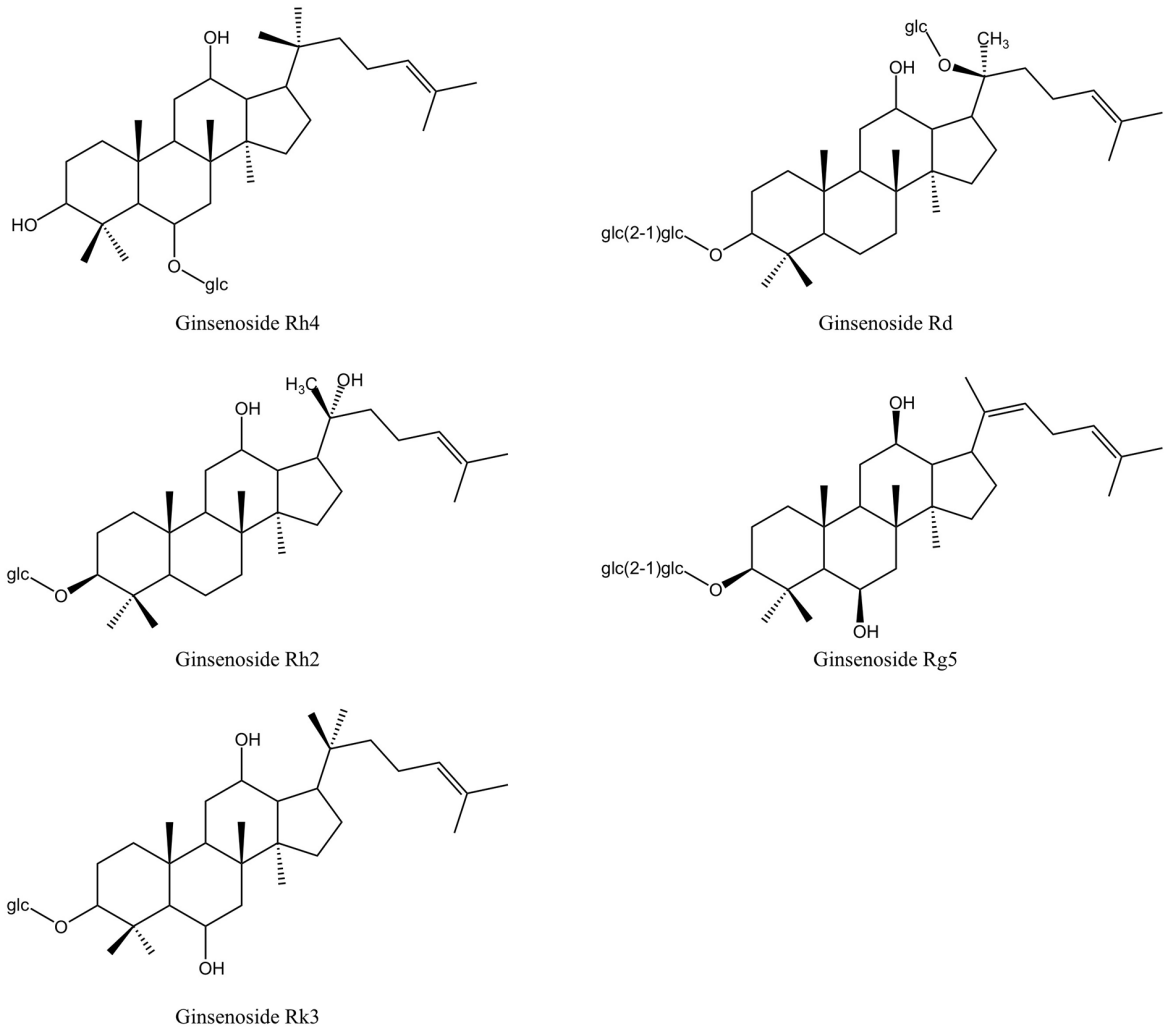
Saponins derived from MFH plants with antibacterial properties (created with KingDraw chemical structure editor).

Saponins are composed of oligosaccharides partially linked with triterpenoids or steroidal aglycones, which can be divided into Triterpenoid saponin and steroidal saponin (82). They are prevalently present in plants as active compounds and have important pharmacological activities. Similar to surfactants, saponins include both hydrophilic and lipophilic groups. By lowering surface tension in aqueous solutions and producing micelles at crucial locations, these chemicals can alter the structure of biological macromolecules (44). Figure 3 displays the saponins from MFH plant sources that can be employed as antibacterial agents.

In China, *Panax ginseng Meyer* (ginseng) has an extended tradition of use in both food and medicinal. Ginsenosides are triterpenoid saponins isolated from ginseng (83). According to research, ginsenosides’ primary antibacterial targets are bacterial cell membranes, and their amphiphilic characteristics are essential for membrane binding and permeability (84). *P. gingivalis* treated with ginsenosides can show the damage of cell membrane system and the outflow of cell contents, and the membrane binding characteristics of ginsenosides also depend on the polarity of ginsenosides, that is, the number of polar sugar groups in the structure (85). High-polarity ginsenosides can be decalcified and dehydrated employing high temperature or biotransformation to reduce polarity and enhance affinity with bacterial cell membranes (44, 86, 87). According to studies, less polar ginsenosides, which contain just one glycosyl group, such as Rk3, Rh2, Rh4 and Rg5, exhibit a better bactericidal action than highly polar ginsenosides, which contain three glycosyl groups and include Rg1, Re, Rb1, Rb2, Rc and Rd. (MIC >500 g/mL). According to TEM images of *F. nucleatum* treated with ginsenoside Rh2, the cell membrane had been destroyed and the contents of the cell had leaked (44). Through experimentation, some researchers have demonstrated that high polar ginsenoside Rd can likewise have a lethal effect on *P. gingivalis* (MIC = 400 μM), but this concentration is quite considerable. Intriguingly, *P. gingivalis* might be limited or even destroyed after treatment with modest concentrations of ginsenoside Rd (100 and 200 μM). Low concentrations of ginsenoside Rd can also drastically reduce bacterial surface hydrophobicity and block the expression of *fimA* and *kgp*, which has an impact on bacterial adhesion, copolymerization, and biofilm formation (45).

### 4.3. Aldehyde

**Figure 4:**
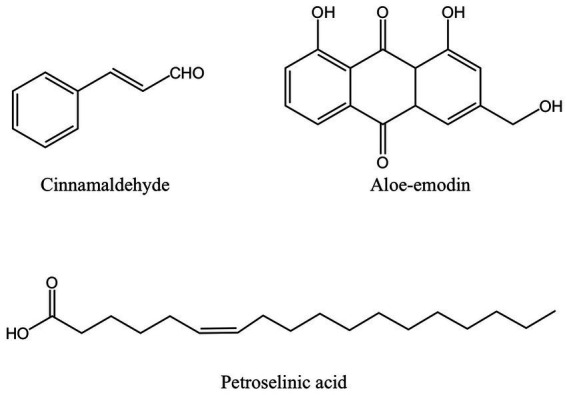
The structure of other antibacterial active ingredients from MFH plants including cinnamaldehyde, aloe-emodin and petroselinic acid (created with KingDraw chemical structure editor).

The dried bark of the cinnamon tree, known as cinnamon, is a common element in Chinese cuisine and traditional herbal remedies. Cinnamaldehyde is an organic compound of olefine aldehydes extracted from cinnamon essential oil (Figure 4) (88). According to several researchers, the main cause of cinnamaldehyde’s antibacterial effects is the destruction of bacterial cell walls and membranes, which causes the contents of the cells to leak out. The interaction of hydrocarbons with hydrophobic bacterial cell membranes is thought to be the cause of this effect, but the antibacterial mechanism is not just restricted to this. It may also be connected to how cinnamaldehyde permeates bacteria and how it affects genetic material (89). In comparison to other chemicals lacking an aldehyde functional group, cinnamaldehyde had the strongest effect on removing *Escherichia coli* biofilm, leading Kot et al. to conclude that the antibacterial action of cinnamaldehyde may be derived from the aldehyde functional group it carries (90). The major component of cinnamon bark essential oil (CBEO), cinnamaldehyde, has been shown in experiments to have a substantial antibacterial impact on the cell wall and membrane of *P. gingivalis*. When *P. gingivalis* was exposed to CBEO and cinnamaldehyde, it developed uneven surfaces, folds, or damage that allowed intracellular proteins, DNA and RNA to leak out. Cinnamaldehyde at the sub-MIC level can inhibit the formation of 67.3% biofilm of *P. gingivalis* (46).

### 4.4. Anthraquinone

*Aloe vera* (aloe) is an edible herb of the lily family. Studies have shown that the gel prepared from mature fresh aloe leaf has an inhibitory effect on pathogens extracted from patients with subgingival stones, periapical abscesses, and periodontal abscesses, but only at a higher concentration. A low concentration of aloe gel does not show an antibacterial effect (91). In other studies, fresh aloe gel has an inhibitory effect on *P. gingivalis*, *A. actinomycetemcomitans*, and other periodontal pathogens, but the effect is not significant compared with *S. mutans* (49). However, both studies lack an analysis of the main active ingredients. When researchers used dried aloe powder extract, the outcomes were very different from those of fresh aloe gel and did not demonstrate any antibacterial activity against *P. gingivalis*. The reason might be that the dried aloe powder contains so little of the anthraquinone component aloe-emodin (Figure 4) (50). Anthracene and two carbonyl groups make form the chemical molecule known as anthraquinone. It is a typical chemical found in numerous plants. Its antibacterial activity primarily consists of impeding DNA synthesis and replication, compromising the integrity of cell membranes, preventing bacterial virulence factors, and preventing the development of biofilm (92–94). However, some academics have also asserted that Aloe-emodin is more likely to attach with peptidoglycan to destroy bacterial membranes and damage cell membrane permeability. Aloe-emodin consequently has more antibacterial effect against gram-positive bacteria than against gram-negative bacteria (95). This may also be one of the reasons why the outcomes of some studies on the bacteriostasis of aloe-emodin on *P. gingivalis* are not ideal. Aloe-emodin’s chemical composition also contributes to its antibacterial properties. According to several researchers, the antibacterial impact is directly tied to the aromatic hydroxyl groups on the anthracene core, and the antibacterial activity is further increased by the hydroxyl methyl groups the compound carries (96).

### 4.5. Fatty acid

*Foeniculum vulgare* (fennel) is a traditional medicinal and culinary plant. Petroselinic acid extracted from fennel is an unsaturated fatty acid (Figure 4). Petroselinic acid induced the formation of moniliform OMVs-like particles on the surface of *P. gingivalis*, which, unlike naturally occurring OMVs, induced explosive degradation of the bacteria and leakage of cell contents and DNA. The accumulation of RagA and RagB in OMVs-like particles can lead to the reduction of RagA and RagB in *P. gingivalis* (55). RagA and RagB proteins are believed to have a part in the uptake of macromolecules like polysaccharides or glycoproteins (97, 98). In addition, fennel extract and petroselinic acid also showed inhibitory effects on gingipains (55). However, the relationship between the anti-*P. gingivalis* and its virulence factor effect of petroselinic acid and its molecular structure has not been elaborated. Some scholars have found that the antibacterial activity of unsaturated fatty acids can be affected by the length of the carbon chain, the degree of unsaturation, and the position and configuration of the double bond. Moreover, the virulence factor and its gene expression of *Staphylococcus aureus* were significantly inhibited by petroselinic acid, suggesting that there may be targets between petroselinic acid and these virulence factors, but this part has not been confirmed by molecular docking research (99).

### 4.6. Others

In addition, some MFH plants have antibacterial activity. Some researchers have proposed that ethanol extract of *Hibiscus sabdariffa* L. (roselle calyx) has inhibitory effects on a variety of periodontal pathogens (48), but this study did not discuss the key active elements or the precise processes of roselle calyx. However, other researchers have hypothesized that protocatechuic acid, which, by generating ROS, damages DNA and causes lipid peroxidation in bacteria, changing the redox equilibrium (100), may be in charge of the roselle calyx’s antibacterial properties (101). More research is necessary to determine whether proto-catechuic acid has the same antibacterial impact against periodontal infections. Ethanol extract of *Morus alba* leaves can reduce the growth of periodontal pathogens in a dose-dependent manner (102). Similarly, diosgenin extracted from *Trigonella foenum-graecum* L. (fenugreek) also showed dose-dependent inhibition of *P. gingivalis* and *P. intermedia* (103). Yet neither study provided information on effective doses.

In conclusion, periodontitis-related bacteria have been found to be significantly inhibited by MFH plants and their active ingredients. In contrast to the direct killing of bacteria, the majority of MFH plants and their active ingredients appear to be more effective at preserving the stability and balance of the periodontal microenvironment by inhibiting bacterial accumulation, copolymerization, biofilm establishment, and the production and activity of bacterial virulence factors. Based on the aforementioned studies, several academics stress the significance of these active components’ chemical structures for their antibacterial activity. Each active ingredient’s antibacterial effect can be attributed to a unique bond or functional group in its chemical structure that can interact and bind with a particular bacterial structure or enzyme, disrupt the physiological function of bacteria result in abnormal bacterial activity or metabolism, and ultimately produce the desired antibacterial effect.

The aforementioned research still has some incomplete aspects in several areas, though. First off, rather than being primarily linked to the pathogenic properties of a specific bacterium, periodontitis tends to be more susceptible to abnormalities in the oral microbial ecosystem. The mouth cavity contains about 1,000 bacteria. Other microbes outside bacteria exist as well, including fungi, viruses, and archaea (104). Distinct strains of microbes can produce distinct active components due to differences in their surface properties and functional groups. Future research should take into account the variations between the harmful bacteria because the aforementioned tests mostly consider one or two of them. In addition, the aforementioned studies did not address whether the active components of some MFH plants can achieve the ideal antibacterial effect *in vivo*, whether the bactericidal effect is particular, or whether it can affect the stability of the overall microenvironment of the oral flora. Therefore, more research is required.

## 5. Medicine food homology plants inhibit host inflammatory response and bone resorption

Although the development of early periodontal tissue lesions requires the presence of bacteria, their significance should not be overstated. As was previously stated, bacteria alone cannot predict how periodontitis will develop. Recent studies have also highlighted the importance of the role of host inflammation and the immune system as opposed to particular bacteria in the etiology of periodontitis. To take part in the inflammatory response, immune cells react to bacterial stimuli by triggering a number of signaling pathways and producing a range of inflammatory cytokines.

The oxidative stress in periodontal tissue and the absorption of alveolar bone may be directly related to the inflammatory response of periodontal tissue. The development of oxidative stress is attributed to an imbalance between the immune system’s capacity to eradicate ROS produced by tissues and the ROS’s actual creation. Some researchers have hypothesized that inflammation and oxidative stress are interconnected and can be mutually causative, causing and boosting one another in terms of their relationship (105). The oxidative stress environment in periodontal tissue, which is created by neutrophil hyperactivity and excessive ROS generation, is critical in causing periodontitis to develop, as was previously discussed. Additionally, the high production and ongoing stimulation of pro-inflammatory cytokines appear to be intimately linked to this excessive activation (106). Therefore, the development of oxidative stress can heighten the periodontal tissue’s inflammatory response, causing substantial harm to the tissue. Similar to this, because of the intricate relationships and dynamic interplay between inflammatory cells and bone cells, inflammatory reactions can significantly alter bone homeostasis and bone remodeling (107). Firstly, cytokines activated during the inflammatory response process, such as IL-1β, IL-6, and TNF- α, and signal pathways, especially nuclear factor kappa B (NF- κβ), have been shown to be closely related to the differentiation and activity of osteoclast, and can promote the process of bone absorption by promoting the differentiation of osteoclast; Secondly, numerous inflammatory mediators can further inhibit the differentiation of osteoblasts and affect bone remodeling. The above two points are important mechanisms for bone mass reduction caused by inflammation (108, 109). In other words, an inflammatory response can undermine the stability of the bone microenvironment, upset the delicate balance between bone creation and absorption, and is inextricably linked to periodontitis’ symptoms of bone absorption. Numerous studies have demonstrated that MFH plant extracts and secondary metabolites can inhibit the production of proinflammatory cytokines, decrease the inflammatory response and oxidative stress of periodontal tissue, and affect the process of osteoclast formation, thereby inhibiting alveolar bone absorption by regulating the aforementioned signal pathways or by altering the transcription and translation of specific regulators (Table 3).

**Table 3:** Inhibitory effects of secondary metabolites derived from the plant MFH on the inflammatory response and bone resorption.

MFH plants	Secondary metabolites	Type of compounds	Related mechanism	Ref.
Cinnamon	Cinnamaldehyde	Aldehyde	Diminishing the *P. gingivalis*-activated NF-κβ signal pathway and reducing the expression of inflammatory factors and ROS generation in RAW264.7 cells. Inhibiting *P. gingivalis*-induced upregulation of pro-inflammatory cytokines in HPDLCs. Downregulation of immune cell chemokines including MCP1, ICAM1, and VCAM1. Promoting the expression of osteogenic differentiation markers in HPDLCs and improving oxidative stress status. Inhibiting the phosphorylation of P65 and Iκβ and inactivating the NF-κβ signaling pathway.	(110)
Psoraleae	Psoralen and angelicin	Phenolic compounds	Blocking the release of IL-1β and IL-8 from monocyte-like THP-1 cells stimulated by *P. gingivalis* LPS. Boosting the expression of osteogenic proteins as well as the activity of alkaline phosphatase in HPDLCs.	(53)
Mulberry	Resveratrol	Phenolic compounds	Inhibiting the activation of transcription factors of the human monoblastic leukemia cell induced by *P. gingivalis*, and interfering with the NF-κβ signal pathway in a dose-dependent manner. Downregulation of the expression of TREM-1 mRNA and reduction of inflammatory response in THP-1 cells induced by *P.gingivalis*. Improving the inflammatory response and oxidative stress of rat periodontal tissue. Decreasing the protein levels of COX-2, MMP-2, and MMP-9 in rats with periodontitis, preventing the reduction of HO-1 and Nrf2 and alveolar bone loss by inhibiting osteoclast production.	(54, 111)
Turmeric	Curcumin	Phenolic compounds	Downregulation of COX-2 mRNA and protein expression in HGFs by inhibiting LPS-activated NF-κβ activation. Counteracting rise of IL-1β and TNF-α induced by LPS in rat gingival fibroblasts. Decreasing the ratio of OPG/RANKL in LPS-induced rat gingival fibroblasts.	(112–114)
Licorice	Glycyrrhizin (Glycyrrhizic acid)	Saponin	Inhibiting the expression of HMGB1, IL-6, and IL-1β in PDLSCs induced by TNF-α. Suppressing the production of HMGB1 and RAGE mRNA in the gingiva and serum and lessening the inflammatory response of the periodontal tissue.	(115, 116)
	Glycyrrhetinic acid	Saponin	Preventing the rise in vascular endothelial permeability brought on by *P. gingivalis* LPS. Impacting vascular endothelial permeability by preventing *P. gingivalis* LPS-promoted the internalization of VE-cadherin of HMECs, which is the cause of the rise in vascular endothelial permeability. Reducing the expression of IL-8, which increases vascular endothelial permeability in HMEC by preventing the activation of NF-κβ.	(117)
	Isoliquiritigenin	Phenolic compounds	Inhibiting the expression level of RANKL-induced osteoclast related genes and transcription factors in RAW264.7 and BMMs, and inhibiting the differentiation of osteoclast. Inhibition of the activation of NF-κβ pathways and blocking the initiation step of osteoclast formation process by inhibiting the binding of RANK and TRAF6. The expression of the c-Fos protein and NFATc1 has also been found to decrease.	(118)
Aloe	Aloe-emodin	Anthraquinone	Down-regulation of AMcase.	(119)
	Aloin	Anthraquinone	Inhibition of phosphorylation of p38 and ERK and downregulation of IL-1β Induced IL-8 expression level.	(120)
Ginseng	Ginsenoside Rd	Saponin	Downregulation of the expression levels of osteoclast marker genes Acp5, Nfatc1 and Mmp9 induced by RANKL to inhibit osteoclast formation, and downregulating the expression levels of inflammatory cytokines such as IL-1β, IL-6, and IL-8 in LPS-induced HGFs. Inhibiting alveolar bone resorption and destruction in mice models of periodontitis.	(45)
	Ginsenoside Rb3	Saponin	Inhibiting the mRNA expression of Nfatc1, Mmp9, Ctsk and Acp5 and decreasing the protein expression levels of MMP-9 and CTSK by inhibiting the RANKL-activated MAPK and NF-κβ signaling pathway in RAW264.7 cells. ERK pathway may be the target of ginsenoside Rb3	(121)
	Ginsenoside Rg1	Saponin	Inhibiting Drp1-mediated mitochondrial fission by activating AMPK and blocking NLRP3-mediated pyroptosis of HPDLCs.	(122)
Papaya	β-cryptoxanthin	Terpenoid	COX-2 and mPGES-1 are downregulated by β-cryptoxanthin in osteoblasts. And β-cryptoxanthin also decreases the NF-κβ transcription activity.	(123)
Ginger	6-Shogaol	Phenolic compounds	Inhibiting the production of ROS in AGEs-induced HGFs, and upregulating the expression levels of two antioxidant factors HO-1 and NQO1. Inhibiting the phosphorylation of MAPK p38, ERK, and NF-κβ p65, as well as the production of pro-inflammatory cytokines. Blocking the MAPK signal transduction caused by RANKL in BMMs.	(124, 125)
*Schisandra chinensis*	Gomisins G and gomisins J	Phenolic compounds	Inhibiting the activation of NF-κβ, downregulating the expression levels of TNF-α, IL-1β and IL-6, and inducing HO-1 production by blocking the nuclear translocation of NF-κβ in RAW264.7 cells induced by *P. gingivalis*	(126)
	α-Iso-cubebenol	Terpenoid	Mediating nuclear translocation and transactivation of Nrf2 in THP-1 cells by promoting the PI3K/Akt and ERK pathways, inducing HO-1 mRNA and protein expression. Inhibiting the nuclear translocation of NF-κβ and its activity. Inhibiting the production of *P.gingivalis* LPS-stimulated pro-inflammatory cytokines.	(127)
	Schisandrin	Phenolic compounds	Enhancing Nrf2 nuclear translocation and HO-1 expression by inducing PI3K/Akt and ERK signaling pathways.	(128)
Pueraria	Puerarin	Phenolic compounds	Suppressing the activation of Akt, downregulating the expression of genes involved in osteoclast formation, and blocking the differentiation of RAW264.7 cells into osteoclasts and the release of proinflammatory substances. Preventing the ubiquitination degradation of Nrf2.	(129, 130)
*Eucommia ulmoides*	Geniposidic acid	Iridoid glycoside	Down-regulating MAPK phosphorylation and TLR2 expression in HGECs induced by *P. gingivalis*, inhibiting IL-6 production. Blocking the expression of genes linked to osteoclast differentiation in BMMC.	(131)
*Magnolia officinalis*	Magnolol	Phenolic compounds	Activation of the Nrf2/HO-1 signaling pathway to reduce the oxidative stress and inflammatory response of RAW264.7 caused by LPS. Inhibiting the expression of COX-2 and iNOS protein in gingival tissue, reduced the ratio of MMP-1/TIMP-1 and MMP-9/TIMP-1.	(132, 133)
Epimedium	Icariin	Phenolic compounds	Increasing OPG in human periodontal cells and downregulated the expression level of RANKL. Increasing the mRNA expression of Cbfa1 and OC. Increasing the protein and gene expression levels of alkaline phosphatase, which is downregulated by LPS in HPDLCs.	(134, 135)

### 5.1. Aldehyde

It has been demonstrated that cinnamaldehyde is a very effective anti-inflammatory medication (136). Cinnamaldehyde was found to diminish the *P. gingivalis*-activated NF-κβ signal pathway, which in turn reduced the expression of inflammatory factors and ROS generation in RAW264.7 cells. The same anti-inflammatory function has also been confirmed in Human periodontal ligament cells (HPDLCs). Cinnamaldehyde inhibits *P. gingivalis*-induced upregulation of pro-inflammatory cytokines in HPDLCs, including IL-6, IL-8, TNF-α and IL-1β. Downregulation of immune cell chemokines including monocyte chemoattractant protein 1 (MCP1), intercellular adhesion molecule 1 (ICAM1) and vascular cell adhesion molecule 1 (VCAM1) is also observed. Meanwhile, cinnamaldehyde also promotes the expression of osteogenic differentiation markers in HPDLCs and improves oxidative stress status. Cinnamaldehyde also inhibits the phosphorylation of P65 and Iκβ and inactivates the NF-κβ signaling pathway (110). According to academics, the impacts of cinnamaldehyde can be summed up as follows: 1. Cinnamaldehyde can chelate metal ions and alter several signaling pathways; 2. The aldehyde functional groups carried by cinnamaldehyde can give hydrogen ions to neutralize free radicals, lowering oxidative stress and inflammatory reactions in tissues (137). Cinnamaldehyde can be given orally to mice with ligation-induced periodontitis to slow the rate of alveolar bone loss and the levels of inflammatory cells and oxidative stress in periodontal tissue (110).

### 5.2. Phenolic compound

**Figure 5:**
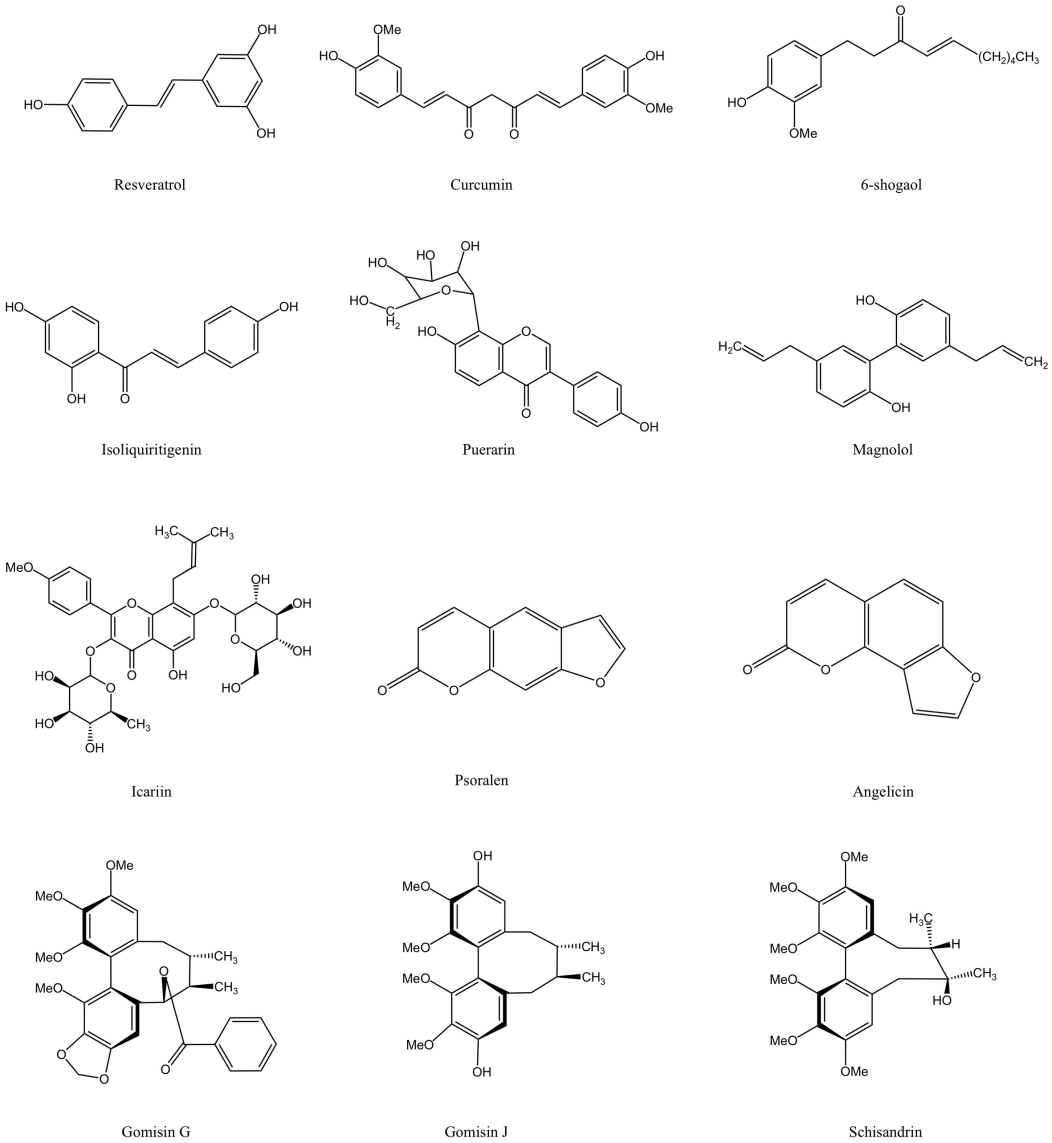
Phenolic compounds derived from MFH plants with anti-inflammatory effects (created with KingDraw chemical structure editor).

Phenolic compounds are electron or hydrogen atom donors with aromatic rings and multiple hydroxyl groups, which can eliminate free radicals through hydrogen ion or proton transfer, inhibit lipid peroxidation, and inhibit the activation of pro-inflammatory cytokine-mediated inflammatory signaling pathways, reducing oxidative stress and inflammatory response (138, 139). They have developed into one of the areas of research that has received a lot of attention lately. MFH plant-derived phenolic compounds with anti-inflammatory, antioxidant, and bone resorption inhibition properties are shown in Figure 5.

*In-vitro* studies have demonstrated that resveratrol blocks the activation of human monoblastic leukemia cell transcription factors induced by *P. gingivalis*, and interfere with the NF-κβ signal pathway in a dose-dependent manner (54). Triggering receptor expressed on myeloid cells-1 (TREM-1) is closely associated with the production of IL-1β and TNF-α that contribute to the persistence of inflammatory pathways (140). Resveratrol significantly down-regulates the expression of TREM-1 mRNA and reduces the inflammatory response in THP-1 cells induced by *P. gingivalis* (54). Toll-like receptor-4 (TLR-4) is an LPS-specific cell sensor that inhibits LPS-induced osteoblast differentiation and bone remodeling (141). Resveratrol down-regulates LPS-stimulated elevated levels of TLR-4 in human gingival fibroblasts (HGFs), thereby reducing the inflammatory response (111). Matrix metalloproteinase (MMP) is a family of zinc-dependent endopeptidases whose main function is to cleave extracellular matrix proteins, reshape tissues and degrade extracellular matrix under physiological and pathological conditions, and is considered to be an important substance involved in periodontal tissue collapse and inflammatory response during the development of periodontitis (142). Cyclooxygenase-2 (COX-2) promotes the disease process of periodontitis by mediating the inflammatory process in periodontal tissues (143). Nuclear factor erythroid 2-related factor 2 (Nrf2) and its downstream anti-invertases, such as heme oxygenase-1 (HO-1), are essential in resisting cellular oxidative stress and down-regulating the inflammatory response (144). Resveratrol has been shown to help reduce the inflammatory response and oxidative stress of rat periodontal tissue *in vivo* studies, decrease the protein levels of COX-2, MMP-2, and MMP-9 in rats with periodontitis, prevent the reduction of HO-1 and Nrf2, and effectively prevent alveolar bone loss by inhibiting osteoclast production (111).

Similarly, curcumin has shown superior anti-inflammation and antioxidant potential *in vitro* and *in vivo*. Curcumin downregulates COX-2 mRNA and protein expression in HGFs by inhibiting LPS-activated NF-κβ activation (112). Based on this, additional researchers have discovered that LPS can greatly upregulate IL-1β and TNF-α in rat gingival fibroblasts, and that curcumin can counteract this rise. In addition, the ratio of osteoprotegerin (OPG)/nuclear factor B receptor activator ligand (RANKL) increased in LPS-induced rat gingival fibroblasts treated with curcumin, and this bone protection feature was also demonstrated in rats with ligation-induced periodontitis (113). To treat rats with LPS-induced periodontitis, some researchers gave them an oral dose of the corn oil carrier that diluted curcumin each day. In periodontal tissue, downregulation of the expression of the IL-6, TNF-α, and prostaglandin E2 (PGE2) synthase genes was seen after 15 days. But curcumin can only block the pathway at a dose of 30 mg/kg (114). Additionally, more investigators discovered that the curcumin gel shell can minimize inflammatory infiltration and restrict bone absorption similarly to tetracyclines when used as an antibacterial supplementary treatment for scaling and root planning (SRP). Contrary to tetracyclines, interestingly, curcumin-treated rats showed iron deposits in their bone trabeculae, suggesting that curcumin may have the ability to encourage bone growth (145).

*Zingiber officinale* Roscoe (ginger) is a kind of herb of the ginger genus. Its rhizome is used as an essential cooking spice in the home and has a high medicinal value. Volatile phenolic compounds 6-Gingerol are the main irritating compounds present in ginger rhizomes. After heat treatment, they are prone to dehydration reactions to form 6-shogaol, which is also the source of the spicy flavor of ginger (146). Hyperglycemia in diabetes patients can induce protein glycosylation, and produce the advanced glycation end products (AGEs) that can damage periodontal tissue by increasing the oxidative stress of periodontal histiocytes and the expression of inflammatory-related factors (147). *In vitro* experiments showed that 6-shogaol can inhibit the generation of ROS in AGE-induced HGFs and upregulate the expression levels of two antioxidant factors, HO-1 and NAD(P)H quinone dehydrogenase 1 (NQO1). In addition, 6-shogaol can also inhibit the phosphorylation of mitogen-activated protein kinases (MAPK) p38, ERK, and NF-κβ p65, as well as the production of pro-inflammatory cytokines (124). Other studies discovered that 6-shogaol blocked the MAPK signal transduction caused by RANKL in mice bone marrow macrophages (BMMs), which prevented the development of mice osteoclasts in a dose-dependent manner. Additionally, this research showed through *in vivo* tests that 6-shogaol can successfully stop bone loss and inflammation in the periodontal tissue of periodontally ligated mice (125).

Licorice isoliquiritigenin is a flavonoid compound. Some studies have suggested that isoliquiritigenin may reduce the levels of expression of RANKL-induced osteoclast-related genes and transcription factors in RAW264.7 and BMMs, and inhibit the differentiation of osteoclasts. And isoliquitigenin inhibits the activation of NF-κβ pathways and blocks the initiation step of the osteoclast formation process by inhibiting the binding of RANKL-stimulated receptor RANK and signal adapter molecule TRAF6. The expression of the c-Fos protein and nuclear factor of activated T cells c1 (NFATc1) has also been found to decrease. Animal studies have also supported isoliquiritigenin’s inhibitory effect on bone resorption. When isoliquiritigenin was administered intraperitoneally to mice, their bone loss was significantly reduced (118).

The dried rhizome of *Pueraria lobata* is a kind of traditional medicinal and edible plant. An isoflavone substance called puerarin is derived from *P. lobata*. Puerarin has been shown to suppress the activation of Akt, downregulate the expression of genes involved in osteoclast formation, and block the differentiation of RAW264.7 cells into osteoclasts caused by LPS and the release of proinflammatory substances. Puerarin can greatly reduce LPS-induced bone loss and the number of osteoclasts in the mice skull in later animal trials (129). According to an intriguing study, signaling pathways connected to Nrf2 may be able to shield periodontal tissue from oxidative stress in hypoxic conditions, preventing damage to the tissue. In typical conditions, Nrf2 can be weakened by Kelch ECH-associating protein 1(Keap1). Nevertheless, p62 and Nrf2 are regulated by a positive feedback loop. By strengthening its association with Keap1, p62 can prevent the degradation of Nrf2 by that protein, and the Akt/ mammalian target of the rapamycin(mTOR)signaling pathway influences the production of p62. Puerarin can indirectly prevent the ubiquitination degradation of Nrf2 mediated by Keap1 and enhance the nuclear translocation of Nrf2 in HPDLCs. It can also upregulate the production of p62 *via* stimulating the Akt/mTOR signaling pathway. Puerarin has additionally demonstrated *in vivo* studies that it can successfully stop alveolar bone loss in animals with periodontitis (130).

*Magnolia officinalis* is a member of the magnolia family whose dried stem bark, root bark and branch bark used in ancient Chinese medicine and can also be incorporated into the diet. Magnolia officinalis is the source of the plant polyphenol known as magnolol. According to studies, magnolol can trigger the Nrf2/HO-1 signaling pathway to reduce the oxidative stress and inflammatory response of RAW264.7 caused by LPS (132). In addition, magnolol significantly reduced the inflammatory response of periodontal tissue and alveolar bone loss and decreased the number of osteoclasts and the expression level of RANKL in experimental periodontitis rats. Magnolol also inhibited the expression of COX-2 and iNOS protein in gingival tissue, reduced the ratio of MMP-1/tissue inhibitor of metalloproteinases-1 (TIMP-1) and MMP-9/TIMP-1, and inhibited the destruction of periodontal tissue (133).

*Epimedium brevicornu* Maxim. (epimedium) is a Ranunculus Berberis plant whose dried leaves are used both medicinally and for food. Icariin is a flavonoid compound that is the main active ingredient in epimedium. 0.01 μg/mL icariin increased OPG in human periodontal cells and downregulated the expression level of RANKL, leading to a decrease in the RANKL/OPG ratio. In addition, icariin also increased the mRNA expression of core binding factor alpha1 (Cbfa1) and osteocalcin (OC) in a dose-dependent manner, and the icariin group at 0.01 μg/mL showed the highest expression, which had a bone-promoting effect; however, when the icariin concentration was greater than 0.1 μg/mL, the expression of Cbfa1 and OC was decreased (134). Icariin can also increase the protein and gene expression levels of alkaline phosphatase, which is downregulated by LPS in HPDLCs (135). Local injection of icariin can attenuate the inflammatory process in minipig periodontitis models, and levels of the pro-inflammatory cytokines IL-1β and IFN-γ were lower in the icariin group than in the control group. In addition, icariin can effectively promote the regeneration of periodontal fibers and bone tissue (148).

*Psoralea corylifolia* (psoraleae) is a traditional plant whose fruits can be eaten and used medicinally. Psoralen and angelicin, which are active components of psoraleae and are coumarin compounds, can, in a dose-dependent manner, block the release of IL-1β and IL-8 from monocyte-like THP-1 cells stimulated by P.gingivalis LPS. Additionally, psoralen and angelica can boost the expression of osteogenic proteins as well as the activity of alkaline phosphatase in HPDLCs. The osteoprotective activities of angelicin on the alveolar bone of periodontitis-affected mice were further supported by this work, although the *in vivo* effects of psoralen remain unaccounted for (53).

*Schisandra chinensis* (Turcz.) Baill is a traditional Chinese medicine in the magnolia family. Its fruit can be used as a medicine or as a daily food. The active ingredient of *Schisandra chinensis* is lignans, including gomisins and schisandrin (149). Gomisins G and J inhibit the activation of NF-κβ, downregulate the expression levels of TNF-α, IL-1β and IL-6, and induce HO-1 production by blocking the nuclear translocation of NF-κβ in RAW264.7 cells induced by *P. gingivalis* (126). HO-1 expression is mainly regulated by the phosphoinositide 3-kinase (PI3K)/Akt pathway. In RAW264.7 cells induced by LPS of *P. gingivalis*, schisandrin can enhance Nrf2 nuclear translocation and HO-1 expression by inducing PI3K/Akt and ERK signaling pathways (128). However, there have been no reports on the *in vivo* anti-inflammatory effects of the active ingredients of Schisandra chinensis mentioned above.

### 5.3. Terpenoid

**Figure 6:**
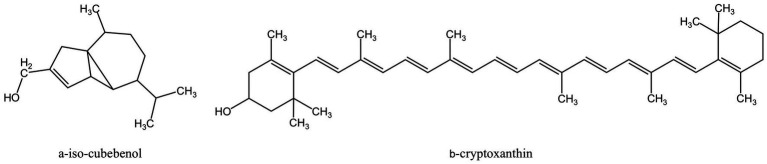
Terpenoids derived from MFH plants with anti-inflammatory effects (created with KingDraw chemical structure editor).

The class of active compounds known as terpenoids, which are abundantly present in nature, is made up of isoprene structural units. They can be categorized as monoterpenes, sesquiterpenes, diterpenes, triterpenes, tetraterpenes, and polyterpenes based on how many isoprene units they contain. Terpenoids have been proven to have significant anti-inflammatory benefits (150). Terpenoids can suppress the development of osteoclasts and the oxidative stress on periodontal tissue as well as operate on a variety of targets in the signal pathway and diminish the generation of inflammatory cytokines. Figure 6 displays the MFH plant terpenoids having anti-inflammatory properties reported in this paper.

α-Iso-cubebenol is a cubebene sesquiterpene compound extracted from *Schisandra chinensis* (151). α-Iso-cubebenol mediated nuclear translocation and transactivation of Nrf2 in human macrophage THP-1 cells by promoting the PI3K/Akt and ERK pathways, induced HO-1 mRNA and protein expression in a time- and dose-dependent manner, and the anti-inflammatory effect of α-Iso-cubebenol can be reversed by the selective inhibitor tin-protoporphyrin of HO-1. In addition, α-Iso-cubebenol can also inhibit the nuclear translocation of NF-κβ and its activity, as well as inhibit the production of *P.gingivalis* LPS-stimulated pro-inflammatory cytokines (127). However, *in-vivo* research has not been done to further support the effects of α-Iso-cubebenol mentioned above.

*Chaenomeles sinensis* (Papaya) is a plant of the genus Papaya in the family of rosaceae. Its fruits are very common in daily life and can be eaten raw or used as a cooking ingredient and, after drying, can also be used as a medicine. β-cryptoxanthin is a carotenoid extracted from Papaya. In LPS-induced mice, β-cryptoxanthin can considerably reduce the amount of cranial bone resorption. Additionally, COX-2 and membrane-bound PGE synthase-1 (mPGES-1), two related enzymes that induce to production of PGE2 in the body, are downregulated by β-cryptoxanthin in osteoblasts. And β-cryptoxanthin also decreases the NF-κβ transcription activity. Molecular docking showed that β-cryptoxanthin competitively binds to the ATP-binding domain of inhibitor of NF-κβ kinase (IKKβ), inhibiting its activity and attenuating the activity of the LPS-induced NF-κβ pathway. In addition, β-cryptoxanthin inhibited RANKL-induced osteoclast differentiation and significantly reduced cathepsin K (CTSK) expression levels in RAW264.7 cells (123).But the effects of β-cryptoxanthin also lack proofs of *in-vivo* experiments.

### 5.4. Iridoid glycoside

**Figure 7:**
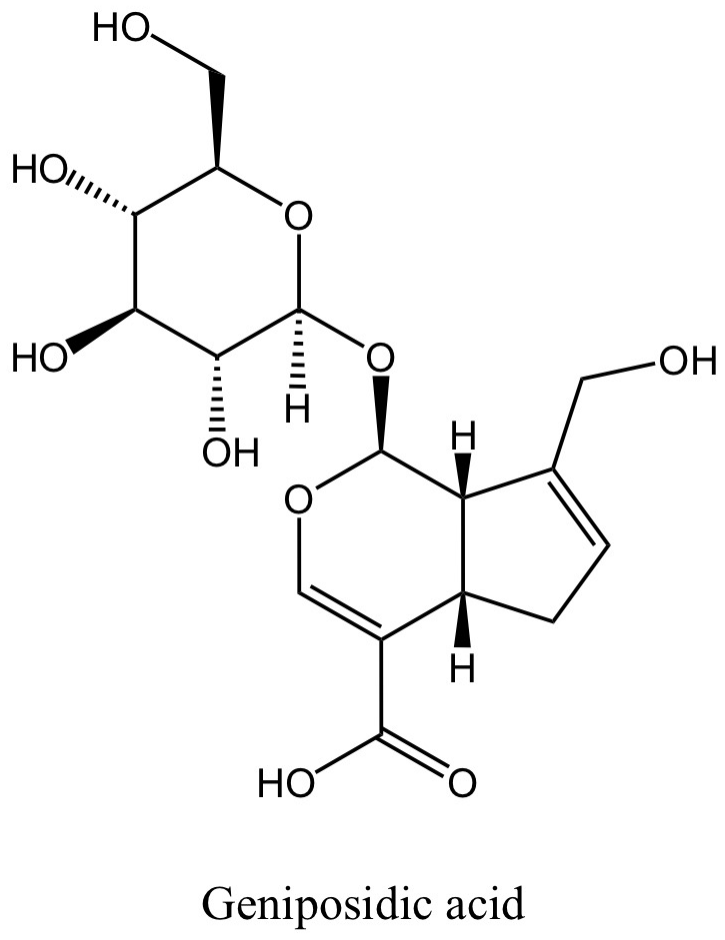
The structure of geniposidic acid (created with KingDraw chemical structure editor).

Iridoid is a natural compound that often combines with sugars in plants to form iridoid glycoside. *Eucommia ulmoides* Oliv. is a plant whose bark is used in traditional medicine and food. Geniposidic acid (Figure 7), an iridoid glycoside extracted from *Eucommia ulmoides*, down-regulates MAPK phosphorylation and TLR2 expression in human gingival epithelial cells (HGECs) induced by *P. gingivalis*, inhibits IL-6 production. Additionally, Geniposidic acid can block the expression of genes linked to osteoclast differentiation in Bone marrow mononuclear cells (BMMC) in a concentration-dependent way. However, Geniposidic acid has no impact on IL-6 levels in BMMC, even though IL-6 is also linked to osteoclast differentiation. Geniposidic acid taken orally can reduce the inflammation of periodontal tissues and alveolar bone absorption in the mice of periodontitis caused by *P.gingivalis* (131).

### 5.5. Anthraquinone

**Figure 8:**
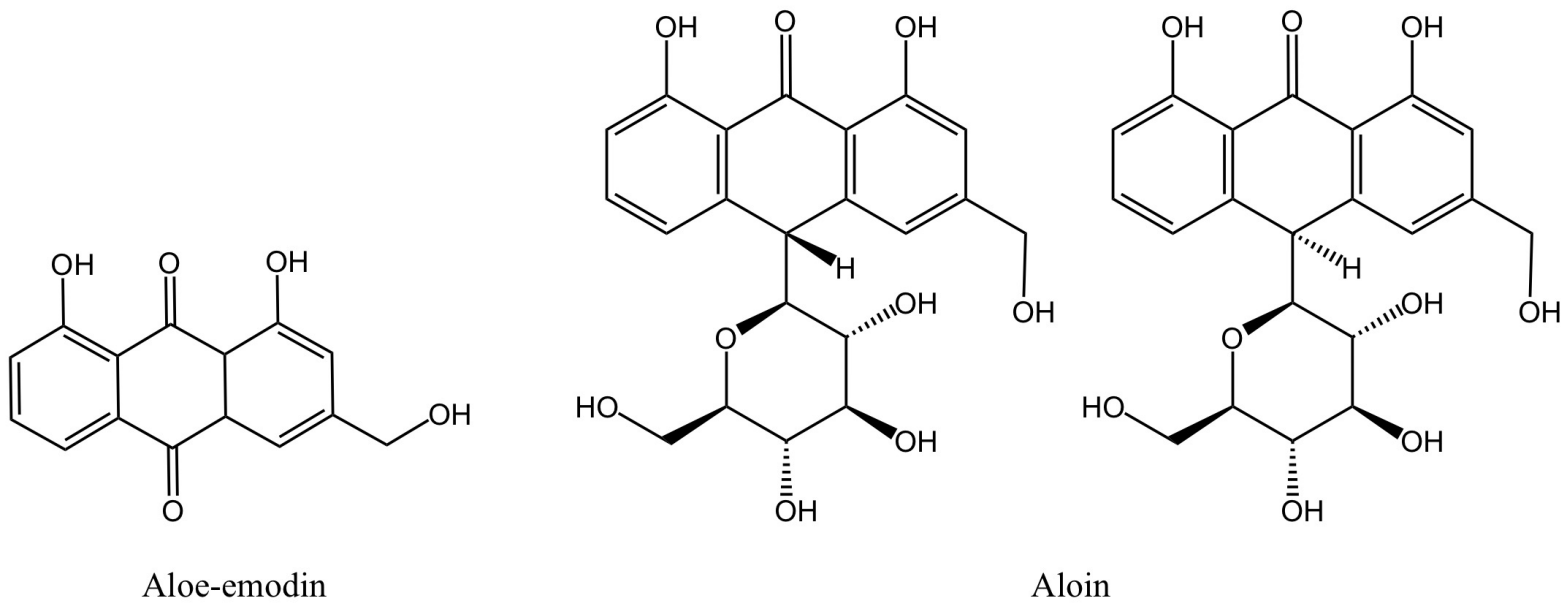
Anthraquinones derived from MFH plants with anti-inflammatory effects (created with KingDraw chemical structure editor).

Both the periodontitis inflammation response and bone resorption are improved by aloe-emodin and aloin derived from aloe (Figure 8). In rat models of periodontitis, aloe emodin has been suggested to lessen alveolar bone loss and periodontal inflammation. The mechanism may be the downregulation of aloe-emodin on acidic mammalian chitinase (AMcase) (119). AMcase is a hydrolase that cleaves glycoside saccharide bonds in chitin. Although humans cannot synthesize chitin, the expression level of AMcase is higher in inflammatory periodontal tissues than in normal periodontal tissues and is associated with the immune response to various inflammatory diseases (152). Aloin is also an anthraquinone compound extracted from aloe, composed of two types of diastereomers (153). Research has shown that aloin down-regulates IL-1β-induced IL-8 expression by inhibiting the phosphorylation of p38 and ERK in KB cells (120), but the anti-inflammatory mechanism *in vivo* has not been elucidated.

### 5.6. Saponin

**Figure 9:**
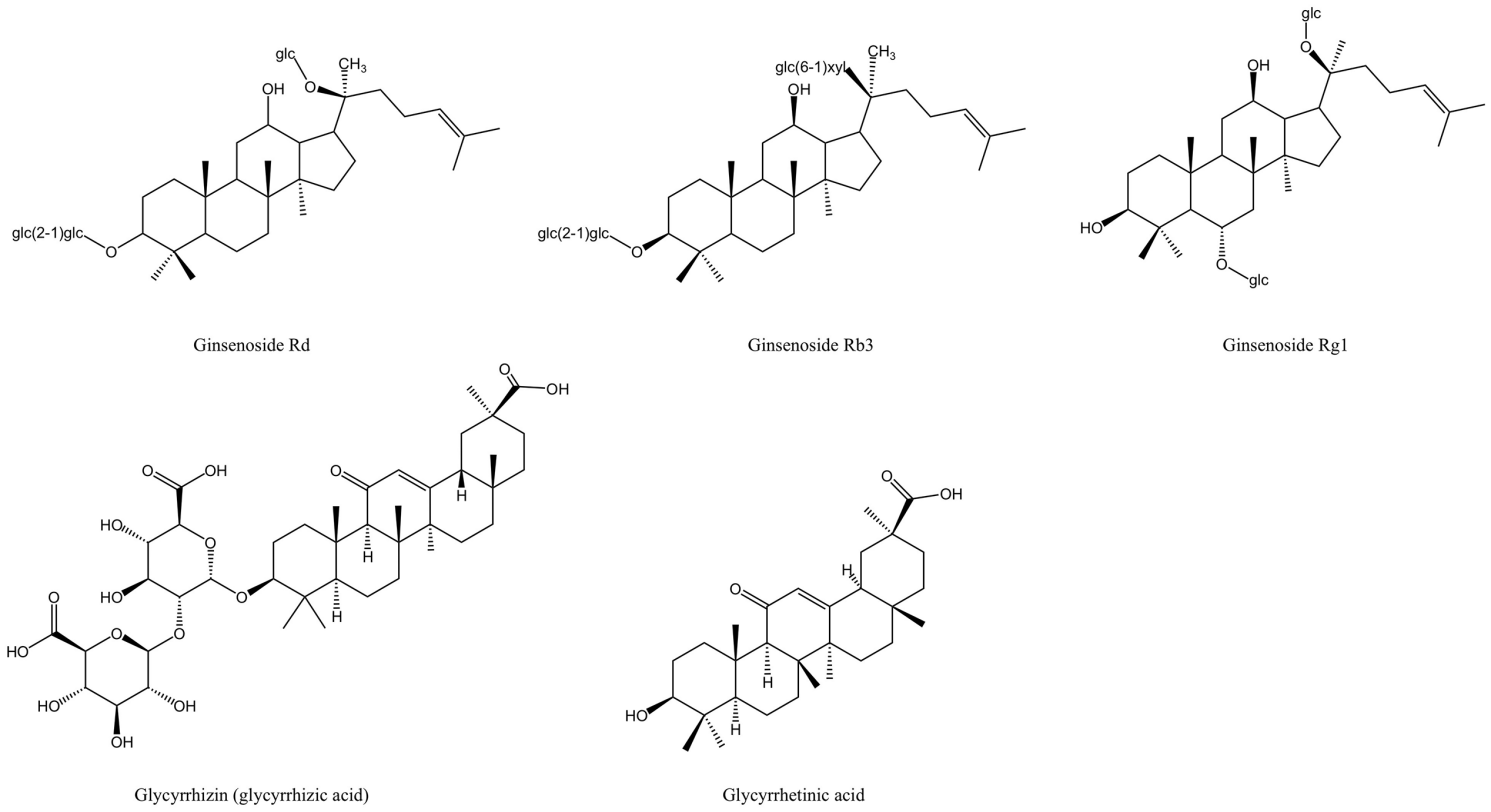
Saponin derived from MFH plants with anti-inflammatory effects (created with KingDraw chemical structure editor).

The saponin components derived from MFH plants also have anti-inflammatory effects (Figure 9). Ginsenoside Rd can downregulate the expression levels of osteoclast marker genes Acp5, Nfatc1 and Mmp9 induced by RANKL to inhibit osteoclast formation *in vitro* and downregulate the expression levels of inflammatory cytokines such as IL-1β, IL-6, and IL-8 in LPS-induced HGFs. Furthermore, *in vivo* experiments have shown that ginsenoside Rd can significantly inhibit alveolar bone resorption and destruction in mice models of periodontitis (45). Ginsenoside Rb3 inhibited the mRNA expression of Nfatc1, Mmp9, Ctsk and Acp5 and decreased the protein expression levels of MMP-9 and CTSK by inhibiting the RANKL-activated MAPK and NF-κβ signaling pathway in RAW264.7 cells. Besides that, ginsenoside Rb3 has the most significant inhibitory effect on the MARK/ERK pathway, suggesting that the ERK pathway may be the target of ginsenoside Rb3 (121). In experimental periodontitis rats stimulated by *P. gingivalis* LPS, Ginsenoside Rb3 inhibits alveolar bone loss and osteoclast differentiation as well (154). Activation of NLRP3 inflammasomes plays an important role in the production of pro-inflammatory cytokines and pyroptosis. Mitochondria are the key regulators of NLRP3 inflammasome activation. Mitochondrial homeostasis is closely linked to AMP-activated protein kinase (AMPK) and dynamin-related protein 1 (Drp1). Ginsenoside Rg1 can inhibit Drp1-mediated mitochondrial fission by activating AMPK, as well as block NLRP3-mediated pyroptosis of HPDLCs in a variety of ways (122). However, the *in-vivo* activity of ginsenoside Rg1 still needs further investigation.

High mobility group protein 1(HMGB1) is a group of non-histone chromosomal proteins that can be released from necrotic or damaged cells. It is highly expressed in periodontitis and can mediate bone resorption by promoting the differentiation of osteoclasts (155, 156). Glycyrrhizin, also known as glycyrrhizic acid, is a triterpenoid saponin isolated from the root of licorice (157). Glycyrrhizin can inhibit the expression of HMGB1, IL-6, and IL-1β in human periodontal ligament stem cells (PDLSCs) induced by TNF-α, according to *in vitro* investigations. *In vivo* test using a rat periodontitis model treated with glycyrrhizin revealed the downregulation of HMGB1, IL-6, and IL-1β in the gingival crevicular fluid and periodontal tissue (115). AGEs receptors (RAGE) in tissues and plasma of diabetic patients bound to HMGB1 are key in the association between periodontitis and diabetes (158). In diabetic mice with periodontitis, glycyrrhizic acid can suppress the production of HMGB1 and RAGE mRNA in the gingiva and serum and lessen the inflammatory response of the periodontal tissue (116). Glycyrrhetinic acid is the main metabolite of glycyrrhizin with two residues of glucose acid (157). According to the *in-vivo* Miles vascular permeability assay, glycyrrhetinic acid can prevent the rise in vascular endothelial permeability brought on by *P. gingivalis* LPS. Studies conducted *in vitro* have demonstrated that glycyrrhetinic acid impacts vascular endothelial permeability by preventing *P. gingivalis* LPS-promoted internalization of VE-cadherin of human microvascular endothelial cells (HMECs), which is the cause of the rise in vascular endothelial permeability. Additionally, by preventing the activation of NF-κβ, glycyrrhetinic acid can reduce the expression of IL-8, which increases vascular endothelial permeability in HMEC (117). Some researchers applied glycyrrhetinic acid locally to the gingival sulcus of the rat periodontitis model and found that glycyrrhetinic acid could effectively inhibit the formation of LPS-stimulated immune complex and inflammatory cell infiltration, and no loss of attachment was observed (159).

### 5.7. Others

In addition to the secondary metabolites from MFH plants outlined above, several MFH plant extracts also have notable inhibiting impacts on periodontal tissue inflammation and bone resorption, such as *Angelica dahurica*, *Ginkgo biloba*, *Houttuynia cordata*, *Chrysanthemum morifolium* Ramat. (chrysanthemum), and *Evodia rutaecarpa* (160–164), although the active biological components they contain have not yet been identified. Table 4 lists the detailed effects of these extracts of MFH plants.

**Table 4:** Inhibitory effects of MFH plant extract on inflammation and bone resorption.

MFH plants	Related mechanism	Ref.
*Angelica dahurica* (Fisch. ex Hoffm.) Benth. & Hook.f. ex Franch. & Sav.	By blocking the expression of NF-κβ and the phosphorylation of Iκβ in LPS-stimulated Raw264.7 cells, *Angelica dahurica* extracts can down-regulate the expression levels of IL-1β, IFN-γ, IL-6 and IL-8. The expression of COX-2 and iNOS was also reduced after treatment with *Angelica dahurica* extract. Furthermore, *in-vivo* studies showed that *Angelica dahurica* extracts significantly improved inflammation and epithelial hyperplasia in rat models of experimental periodontitis.	(160)
*Ginkgo biloba*	The extract of its dried leaves can inhibit the activation of the MAPK pathway in RAW264.7 cells induced by *P.gingivalis* LPS, block the nuclear translocation of NF-κβ and activator protein-1. The expression levels of the pro-inflammatory cytokines TNF-α, IL-1β and IL-6 are expressed at lower levels in response to the extract of its dried leaves. In addition, the extract of *Ginkgo biloba* also induced the expression of HO-1 by improving the binding activity between Nrf2 and the ARE sequence of the HO-1 promoter region.	(161)
*Houttuynia cordata*	Its extract down-regulates the expression levels of pro-inflammatory cytokines IL-8, ICAM-1 and MMP-3 and their related genes by inhibiting the phosphorylation of ERK in HGECs induced by *A. actinomycetemcomitans.*	(162)
Chrysanthemum	By reducing the expression of osteoclast differentiation marker genes as well as the phosphorylation of MAPK p38 and JNK, chrysanthemum water extract can have an impact on osteoclast formation in RANKL-stimulated BMMs. Additionally, chrysanthemum water extract can reduce the expression of c-Fos and NFATc1, important transcription factors in osteoclast development, as well as the activation of phospholipase C gamma 2 and cAMP response element-binding proteins linked to osteoclast differentiation.	(163)
*Evodia rutaecarpa*	By inhibiting IL-1β-induced MAPK/ signal transducer and activator of transcription 3 activation in HGFs, the extract of the fruits of *Evodia rutaecarpa* significantly inhibited the expression of MMP-1 and MMP-3. In addition, the expression levels of the pro-inflammatory stimulators TNF-α, IL-8 and IL-6 were also downregulated.	(164)

In conclusion, there is considerable biological activity and minimal toxicity in the extracts and secondary metabolites of MFH plants against periodontal tissue inflammation. Although there has been a substantial amount of *in vitro* and animal experimentation demonstrating the majority of the anti-inflammatory and anti-bone resorption benefits of MFH plants, clinical research is still in its infancy. Additionally, it is a difficult and time-consuming operation to identify and analyze the active components in some research that focus on extracts of MFH plants. There are still a lot of undiscovered linkages that need to be investigated, but some researches have established a causal link between the chemical makeup of some active chemicals and their anti-inflammatory and bone resorption action. However, the number of such studies is too limited. Furthermore, some researches indicate that the dosage or concentration of MFH plants is strongly related to their biological action, and high or low doses may significantly decrease or boost the biological activity of the active ingredient, such as large quantities of phenolic compounds that can cause oxidation (138). And different active component dosages may have various modes of action. For instance, various dosages of curcumin can influence various signal pathways and prevent the creation of certain pro-inflammatory substances (114). Therefore, dose control still requires special care from us.

## 6. Evidence from clinical studies of MFH plants

The number of clinical investigations is still extremely small, although some MFH plants have demonstrated considerable anti-periodontitis benefits *in vitro* and animal tests. The ability of MFH plants to enhance periodontal health in individuals with periodontitis has been validated by certain researchers, however, the majority of the studies have been modest in scope and substance. The present research and development philosophy for MFH plant products is centered on clinical adjuvant therapies, medicines and functional foods.

### 6.1. Medicine food homology plants related periodontal adjuvant therapy

Plant-based MFH products are frequently used in conjunction with periodontal therapy to help keep the therapeutic impact going strong. Babaei et al. (165) assigned 40 patients with chronic periodontitis to intervention and control groups and administered 1 g of *Cichorium intybus* L. (chicory) leaf methanol extract capsules and placebo capsules, respectively, twice daily for consecutive 8 weeks. Before the study, all subjects underwent non-surgical periodontal therapy. According to the findings, chicory leaf extract was able to significantly improve the depth of the periodontal pocket, increase total antioxidant capacity, uric acid and high-density lipoprotein cholesterol and decrease malondialdehyde, triglycerides, low-density lipoprotein cholesterol and total cholesterol levels in the intervention group compared to the control group and baseline (*p* < 0.05).

### 6.2. Medicine food homology plants related functional foods and oral care products

Twenty young adults with healthy periodontal conditions were recruited by Gao et al. (166) and divided into an intervention group and a control group before being given either *Phyllanthus emblica* L. gum or a placebo gum. It turns out that in the group of *P. emblica* gum, the generation of volatile sulfide was inhibited, and the overall bacterial count, *S. mutans*, and *P. gingivalis* counts were significantly reduced (*p* < 0.05). Assiry et al. (167)recruited 50 subjects with moderate gingival scores and fair plaque scores without systemic diseases and randomly assigned them to the intervention and control groups. The intervention group received *Illicium verum* (star anise) mouthwash, whereas the control group received colored placebos, twice daily. Results indicate that subjects in the *I. verum* intervention group significantly improved in mean gingival index, papillary bleeding index and microbial count (*P* 0.05) when compared to the control group and baseline (*p* < 0.05).

In summary, MFH plant-based functional foods and care items such as chewing gum, chewing tablets, mouthwash, toothpaste, and so on need to be enhanced in terms of flavor and taste while ensuring effectiveness, as adding MFH plant-based ingredients may result in an unpleasant odor and taste. How to strike the right balance between the degree of delicacy and the optimal clinical effect is one of the issues that need to be resolved. Future research should also take into account the low solubility issue with some active compounds generated from MFH plants. This issue can be resolved by developing a scientifically sound drug delivery system that will prolong the action duration inside the periodontal tissue and decrease drug loss.

## 7. Conclusion

The following might be used to summarize how MFH plants work to treat and prevent periodontitis: First, bacteria are the primary causes of inflammation. Bacterial cell membranes are susceptible to the effects of MFH plants and their secondary metabolites, which can cause bacterial cell disruption and expulsion of cell contents. They may also hinder the synthesis of biological macromolecules vital to bacterial survival at the same time, impairing bacterial growth and reproduction; Second, by preventing the action of bacterial virulence factors and the expression of associated genes, MFH plants and their secondary metabolites can also affect bacterial invasion, colonization, copolymerization, biofilm formation, and periodontal tissue damage; Additionally, MFH plants can lessen bone absorption by limiting the development of osteoclasts and can lower the level of inflammatory cytokines in periodontal tissue by controlling the synthesis of a range of signal pathways and regulatory variables; Last but not least, the modulation of antioxidant macromolecules, the downregulation of the oxidative stress response in periodontal tissue, and the lessening of damage to periodontal tissue are all possible functions of MFH plants. Significant progress has been made in the study of the use of MFH plants in treating periodontitis, which is encouraging for the creation of new MFH plant-related functional meals, oral care products, or additional methods of treating periodontitis.

Periodontitis, as a worldwide public health problem, mainly manifests as gingival bleeding and retraction, alveolar bone loss, bad breath, and so on. In extreme circumstances, it can lead to tooth loss and affect the patient’s quality of life. Periodontitis is currently mostly treated with clinically necessary care and antibacterial adjuvant therapy. However, the development of multidrug-resistant bacteria brought on by antibiotics has long been a challenge that many academics are working to overcome. Therefore, one of the major research hotspots is the hunt for plant-based medicines that can replace antibiotics. The fact that variables other than oral ones can promote inflammation in periodontal tissue adds to the complexity of the etiology of periodontitis. It is undeniable that some systemic conditions, including diabetes and obesity, have strong associations with periodontitis. Dietary intervention is one of the most crucial treatment modalities for chronic disorders. The response to the question of whether a balanced diet may help with the prevention and treatment of periodontitis brought on by diabetes or obesity has been raised by a number of academics, and it is clear that it can. Consuming wholesome, anti-inflammatory foods and changing one’s dietary habits have been demonstrated to help treat periodontitis in numerous clinical investigations. In keeping with the new idea of contemporary illness prevention, MFH plants have emerged as the ideal option for disease prevention and a balanced diet due to their dual usage as food and medicine. In conclusion, MFH plants are rich resources that should be further explored to support the clinical conversion of MFH plant-related products. For the future prevention and treatment of periodontitis, they are extremely promising natural products.

## Author contributions

SQ: conceptualization, visualization, and writing–original draft preparation. SY: conceptualization and writing–review and editing. XM: writing–original draft preparation. RW: supervision, validation, and writing–review and editing. All authors have read and agreed to the published version of the manuscript.

## Funding

This research was funded by “Jilin Health Science and Technology Capacity Improvement Project, 2021JC029” and “Jilin Health Research Project, 2021JK02.”

## Conflict of interest

The authors declare that the research was conducted in the absence of any commercial or financial relationships that could be construed as a potential conflict of interest.

## Publisher’s note

All claims expressed in this article are solely those of the authors and do not necessarily represent those of their affiliated organizations, or those of the publisher, the editors and the reviewers. Any product that may be evaluated in this article, or claim that may be made by its manufacturer, is not guaranteed or endorsed by the publisher.
